# Glucocorticoid Excess in Patients with Pheochromocytoma Compared with Paraganglioma and Other Forms of Hypertension

**DOI:** 10.1210/clinem/dgaa423

**Published:** 2020-07-01

**Authors:** Georgiana Constantinescu, Katharina Langton, Catleen Conrad, Laurence Amar, Guillaume Assié, Anne-Paule Gimenez-Roqueplo, Anne Blanchard, Casper K Larsen, Paolo Mulatero, Tracy Ann Williams, Aleksander Prejbisz, Martin Fassnacht, Stefan Bornstein, Filippo Ceccato, Stephanie Fliedner, Michael Dennedy, Mirko Peitzsch, Richard Sinnott, Andrzej Januszewicz, Felix Beuschlein, Martin Reincke, Maria-Christina Zennaro, Graeme Eisenhofer, Jaap Deinum

**Affiliations:** 1 Department of Medicine III, University Hospital Carl Gustav Carus, Technische Universität Dresden, Dresden, Germany; 2 Grigore T. Popa University of Medicine and Pharmacy, Iasi, Romania; 3 Institute of Clinical Chemistry and Laboratory Medicine, University Hospital Carl Gustav Carus, Technische Universität Dresden, Dresden, Germany; 4 Hôpital Européen Georges Pompidou, Hypertension Unit, APHP, Paris, France; 5 Cardiovascular Research Center INSERM, Paris, France; 6 Paris Descartes University, Sorbonne Paris Cité, Paris, France; 7 Department of Endocrinology, Center for Rare Adrenal Diseases, Hôpital Cochin, APHP, Paris, France; 8 Institut Cochin, INSERM, Paris, France; 9 Hôpital Européen Georges Pompidou, Genetics Unit, Paris, France; 10 INSERM, Centre d’Investigations Cliniques, Paris, France; 11 Division of Internal Medicine and Hypertension, Department of Medical Sciences, University of Turin, Italy; 12 Medizinische Klinik und Poliklinik IV, Klinikum der Universität München, Munich, Germany; 13 Department of Hypertension, Institute of Cardiology, Warsaw, Poland; 14 Department of Internal Medicine I, Division of Endocrinology and Diabetes, University Hospital, University of Würzburg, Würzburg, Germany; 15 Comprehensive Cancer Center Mainfranken, University of Würzburg, Würzburg, Germany; 16 Division of Diabetes & Nutritional Sciences, Faculty of Life Sciences & Medicine, King’s College London, London, UK; 17 Klinik für Endokrinologie, Diabetologie und Klinische Ernährung, University Hospital, Zürich, Switzerland; 18 Endocrinology Unit, Department of Medicine DIMED, Padova University Hospital, Padua, Italy; 19 First Department of Medicine, University Medical Center Schleswig-Holstein, Lübeck, Germany; 20 The Discipline of Pharmacology and Therapeutics, School of Medicine, National University of Ireland Galway, Galway, Ireland; 21 School of Computing and Information Systems, University of Melbourne, Melbourne, Australia; 22 Department of Internal Medicine, Radboud University Medical Center, Nijmegen, the Netherlands

**Keywords:** pheochromocytoma, paraganglioma, hypertension, steroids, metanephrines

## Abstract

**Context:**

Catecholamines and adrenocortical steroids are important regulators of blood pressure. Bidirectional relationships between adrenal steroids and catecholamines have been established but whether this is relevant to patients with pheochromocytoma is unclear.

**Objective:**

This study addresses the hypothesis that patients with pheochromocytoma and paraganglioma (PPGL) have altered steroid production compared with patients with primary hypertension.

**Design:**

Multicenter cross-sectional study.

**Setting:**

Twelve European referral centers.

**Patients:**

Subjects included 182 patients with pheochromocytoma, 36 with paraganglioma and 270 patients with primary hypertension. Patients with primary aldosteronism (n = 461) and Cushing syndrome (n = 124) were included for additional comparisons.

**Intervention:**

In patients with PPGLs, surgical resection of tumors.

**Outcome measures:**

Differences in mass spectrometry–based profiles of 15 adrenal steroids between groups and after surgical resection of PPGLs. Relationships of steroids to plasma and urinary metanephrines and urinary catecholamines.

**Results:**

Patients with pheochromocytoma had higher (*P* < .05) circulating concentrations of cortisol, 11-deoxycortisol, 11-deoxycorticosterone, and corticosterone than patients with primary hypertension. Concentrations of cortisol, 11-deoxycortisol, and corticosterone were also higher (*P* < .05) in patients with pheochromocytoma than with paraganglioma. These steroids correlated positively with plasma and urinary metanephrines and catecholamines in patients with pheochromocytoma, but not paraganglioma. After adrenalectomy, there were significant decreases in cortisol, 11-deoxycortisol, corticosterone, 11-deoxycorticosterone, aldosterone, and 18-oxocortisol.

**Conclusions:**

This is the first large study in patients with PPGLs that supports in a clinical setting the concept of adrenal cortical–medullary interactions involving an influence of catecholamines on adrenal steroids. These findings could have implications for the cardiovascular complications of PPGLs and the clinical management of patients with the tumors.

Pheochromocytoma is a tumor arising from chromaffin cells of the adrenal medulla that produces the catecholamines epinephrine and norepinephrine in variable amounts (1). When similar tumors arise from extra-adrenal chromaffin cells they are termed paragangliomas and produce mainly norepinephrine and sometimes dopamine.

Hypertension in patients with pheochromocytoma and paraganglioma (PPGL) is considered to mainly result from the vasopressor effects of increased circulating concentrations of norepinephrine and epinephrine. Steroids such as aldosterone and cortisol that originate from the adrenal cortex also increase blood pressure but by different mechanisms such as increased sodium reabsorption in the kidney and actions on the systemic vasculature (2).

An impact of the adrenal cortex on adrenal medullary catecholamine synthesis is well established, starting with observations of over 50 years ago that adrenal synthesis of epinephrine depends on glucocorticoid-mediated induction of phenylethanolamine-N-methyltransferase (PNMT), the enzyme that converts norepinephrine to epinephrine (3). Differences in PPGLs that produce epinephrine versus those that produce predominantly norepinephrine have since been shown to reflect absence or presence of pseudohypoxia-induced blockade of glucocorticoid-mediated induction of PNMT (4). Although impact of adrenal cortex-derived steroids on adrenal medullary catecholamine systems is well established, it has also become apparent that catecholamines reversely impact steroidogenesis, possibly through paracrine actions (5-8). Moreover, besides short-term regulation by catecholamines there is also a long-term effect involving transcriptional regulation of steroid enzyme expression (9). It thus seems that the adrenal medullary and cortical systems are intimately connected (10, 11).

The bidirectional connections between the adrenal cortex and medulla raise the question whether increased production of catecholamines in patients with PPGLs might influence adrenal steroidogenesis. We therefore hypothesized that high production of catecholamines in patients with PPGLs may result in alterations in adrenal cortical steroid systems. To explore this hypothesis we applied a mass spectrometry–based method to measure 15 adrenal steroids in plasma of a cohort of patients with PPGLs compared with additional patients with primary hypertension. For additional comparative purposes we also included in the analysis groups of patients with primary aldosteronism and Cushing syndrome.

## Material and Methods

### Patient recruitment

This retrospective cross-sectional observational study included 1073 participants recruited under a multicenter protocol (Prospective Monoamine-producing Tumor study) and a registry of the European Network for the Study of Adrenal Tumors. Five groups of patients were recruited: pheochromocytoma (n = 182), paraganglioma (n = 36), primary aldosteronism (n = 461), Cushing syndrome (n = 124), and hypertensive volunteers (n = 270). Patients were enrolled from 12 European centers: (1) University Hospital Carl Gustav Carus Dresden, Germany; (2) University Hospital of Munich, LMU, Germany; (3) Hôpital Européen Georges Pompidou, Unité d’hypertension, Paris, France; (4) Hôpital Cochin, Service d’endocrinologie, Paris, France; (5) Cardiovascular Research Center INSERM, Paris; (6) University Hospital of Turin; (7) Institute of Cardiology, Warsaw, Poland; (8) University Hospital of Würzburg, Germany; (9) University Medical Centre Schleswig-Holstein, Lübeck, Germany; (10) Radboud University Medical Centre, Nijmegen, the Netherlands; (11) University Hospital of Padova; (12) University Hospital Galway. Diagnosis was based on results of conventional diagnostic testing following current guidelines (12-14). All study protocols under which patients were recruited were approved by the local ethics committee of each participating center and all subjects provided written informed consent before participation in protocols.

### Plasma steroid profiling

All blood samples for plasma steroid profiling were collected in the morning (08.00-11.00) into blood tubes containing lithium heparin or ethylenediamine tetra-acetate. Separated plasma was stored at –80°C until the steroid profile was analyzed by liquid chromatography tandem mass spectroscopy (LC-MS/MS). All measurements of steroids were performed at a single laboratory in Dresden. The panel included 15 steroids: cortisol, 11-deoxycortisol, 21-deoxycortisol, corticosterone, 11-deoxycorticosterone, aldosterone, 18-oxocortisol, 18-hydroxycortisol, cortisone, progesterone, 17-hydroxyprogesterone, pregnenolone, androstenedione, dehydroepiandrosterone (DHEA), and DHEA sulfate (DHEAS). Full details of the method, including the assay performance characteristics, have been described elsewhere (15).

### Plasma steroid profiling before and after adrenalectomy

Among the patients with PPGLs, additional plasma samples were available from 100 patients at an interval between 12 and 36 months after surgical removal of tumors (88 with pheochromocytoma and 12 with paraganglioma). Thus, steroid profiles for these patients were obtained in paired samples of plasma at screening before surgical intervention and then again after resection of catecholamine-producing tumors.

### Catecholamines and metanephrines

Biochemical measurements of urinary catecholamines and catecholamine metabolites (metanephrine, normetanephrine) in both urine and plasma were performed at a single laboratory in Dresden using LC-MS/MS as described previously (16, 17). Details for blood and urine collections and reference intervals have been described in detail elsewhere (18).

### Statistical analysis

Statistical analyses were carried out using JMP statistics software package (SAS Institute Inc, Cary, NC). Due to non-normal distribution of data the nonparametric Kruskal–Wallis and the Steel–Dwass all pairs tests were used for comparisons involving multiple groups. Spearman’s rank correlation was used to assess significance of relationships. Significance was defined as *P* < .05. Reference intervals for steroids depend on age and sex (19). For additional parametric statistical analyses all data were first logarithmically transformed to normalize distributions and corrected for age and sex using a multivariate model. Multivariate analysis with age and sex as covariates was then carried out to establish significance between groups using the Tukey Honestly significant difference (HSD) post hoc test. Final display of data was achieved from the exponents of least square means to derive geometric means with respective plus and minus standard errors as described elsewhere (20). Within group changes from pre- to postadrenalectomy were calculated using the Wilcoxon signed rank test. The steroids of interest are represented as mean of percentage (%) change with 95% confidence interval (CI), calculated after logarithmic transformation of the fold change and the results retransformed (antilog) as percentages. Graphics were designed using PRISM 8 (Version 8.2.1) and Excel 2016.

## Results

### Patient characteristics

There were equal distributions of sexes in all groups except for patients with Cushing syndrome, in whom the proportions of females were higher (*P* < .0001) at 77% than in the other 4 groups in whom proportions varied from 41% to 56% (Table 1). Patients with paraganglioma were younger (*P* < .05) than those with pheochromocytoma and primary aldosteronism.

**Table 1.** Age and sex distribution

Group	PHT	PGL	PHEO	PA	CS
N	270	36	182	461	124
Sex (F/M)	120/150	17/19	109/73	188/273	95*/29
Age ± SD	49.2 ± 13.3	44. ± 14.4* ^*ab*^	50.0 ± 15.0	51.1 ± 10.6	48.5 ± 15.5

Ages are shown as means** ± **standard deviation (SD).

*P < .005, ^*a*^different from PA, ^*b*^different from pheo.

### Plasma steroids

As shown in Table 2, plasma concentrations of cortisol, 11-deoxycortisol, corticosterone, and 11-deoxycorticosterone were all higher (*P* < .05) in patients with pheochromocytoma than in patients with primary hypertension. In contrast, 18-hydroxycortisol, DHEAS, androstenedione, pregnenolone, and progesterone were lower (*P* < .05) in patients with pheochromocytoma than in patients with primary hypertension. There were also differences between patients with pheochromocytoma and paraganglioma, in whom plasma concentrations of cortisol (*P* = .0010), 11-deoxycortisol (*P* = .032), and corticosterone (*P* = .015) were higher in those with adrenal than extra-adrenal tumors. DHEA and DHEAS were the only steroids elevated in patients with paraganglioma compared with pheochromocytoma (*P* < .05).

**Table 2.** Plasma concentrations of 15 adrenal steroids in the reference group (PHT) compared with patients with paraganglioma (PGL), pheochromocytoma (PHEO), primary aldosteronism (PA), and Cushing syndrome (CS)

	Reference Group	Endocrine hypertension			
Plasma steroids (nmol/L)	PHT	PGL	PHEO	PA	CS
Cortisol	257	248	338*** ^*ab*^	317*** ^*a*^	496*** ^*f*^
	(205-341)	(213-378)	(259-452)	(227-412)	(390-636)
11-Deoxycortisol	0.47	0.47	0.86*** ^*ab*^	0.88*** ^*a*^	1.53*** ^*f*^
	(0.29-0.71)	(0.29-0.93)	(0.45-1.55)	(0.54-1.61)	(0.86-2.85)
21-Deoxycortisol	0.035	0.029	0.043	0.066*** ^*abd*^	0.052* ^*ab*^
	(0.017-0.069)	(0.017-0.049)	(0.020-0.089)	(0.028-0.188)	(0.020-0.121)
Aldosterone	0.121	0.127	0.142	0.354*** ^*f*^	0.093
	(0.068-0.190)	(0.060-0.219)	(0.063-0.260)	(0.201-0.610)	(0.034-0.239)
Corticosterone	4.70	4.20* ^*c*^	6.97*** ^*ab*^	6.61*** ^*a b*^	10.29^***ae*bc^
	(3.05-8.41)	(2.72-7.38)	(4.19-11.82)	(3.62-12.15)	(6.28-17.42)
11-Deoxycorticosterone	0.102	0.154* ^*a*^	0.196*** ^*a*^	0.218* ^*abc*^	0.207^***a^
	(0.052-0.184)	(0.096-0.223)	(0.098-0.281)	(0.146-0.345)	(0.134-0.383)
18-Oxocortisol	0.022	0.027	0.027	0.053^***f^	0.030
	(0.013-0.037)	(0.016-0.069)	(0.016-0.058)	(0.026-0.230)	(0.011-0.047)
18-Hydroxycortisol	1.60	1.26* ^*a*^	1.34^*a^	2.12*** ^*abc*^	2.16*** ^*abc*^
	(1.11-2.28)	(0.83-1.66)	(0.89-1.98)	(1.28-4.01)	(1.21-3.53)
Cortisone	49.52	49.09	52.98	49.11* ^*c*^	55.70* ^*ae*^
	(42.93-58.61)	(42.50-62.85)	(44.05-62.69)	(39.12-59.23)	(42.67-70.02)
DHEA	8.37	11.88^*a^	8.40* ^*b*^	7.76* ^*b*^	6.25
	(5.30-13.90)	(6.73-19.59)	(4.14-13.32)	(4.19-12.5)	(2.03-15.98)
DHEAS_(micomol__)_	8.31	7.63	4.09*** ^*ab*^	5.41^*a^	5.82
	(4.61-12.00)	(4.80-9.27)	(2.24-7.78)	(3.24-8.48)	(1.45-13.80)
Androstenedione	2.97* ^*d*^	2.32	2.23*** ^*a*^	2.69* ^*c*^	3.83* ^*f*^
	(2.19-4.15)	(1.85-2.98)	(1.51-3.19)	(1.78-4.03)	(1.95-7.73)
Pregnenolone	3.75	3.89	3.17^***a^	2.12* ^*ab*^	4.69 ^*e^
	(2.41-6.01)	(1.77-8.27)	(1.09-5.70)	(0.66-6.29)	(1.27-10.00)
17-Hydroxyprogesterone	1.70	1.93	1.41	2.01*** ^*ac*^	1.41* ^*e*^
	(0.58-2.60)	(1.16-3.28)	(0.65-2.47)	(1.05-3.05)	(0.74-2.71)
Progesterone	0.25	0.23	0.12*** ^*f*^	0.25*** ^*c*^	0.23* ^*c*^
	(0.18-0.36)	(0.07-0.48)	(0.05-0.23)	(0.13-0.50)	(0.08-0.51)

Plasma concentrations are shown as medians in nmol/L, the 25th and 75th quartiles are in parentheses.

*P < 0.005, ***P < 0.0001; ^*a*^different from PHT, ^*b*^different from PGL, ^*c*^different from PHEO, ^*d*^different from CS, ^*e*^different from PA, ^*f*^different from all groups.

To account for potential confounding influences of age and sex, we used multivariate analysis to clarify and correct for potential impacts of these variables on the aforementioned differences in plasma steroids between the 5 groups of subjects (all supplementary material and figures are located in a digital research materials repository (21)). This analysis showed negative relationships (*P* < .01) of age with all steroids except 18-hydroxycortisol and 21-deoxycortisol. There were also variable sex differences, which were particularly pronounced for DHEAS, 17-hydroxyprogesterone, and progesterone (*P* < .001).

**Figure 1.**
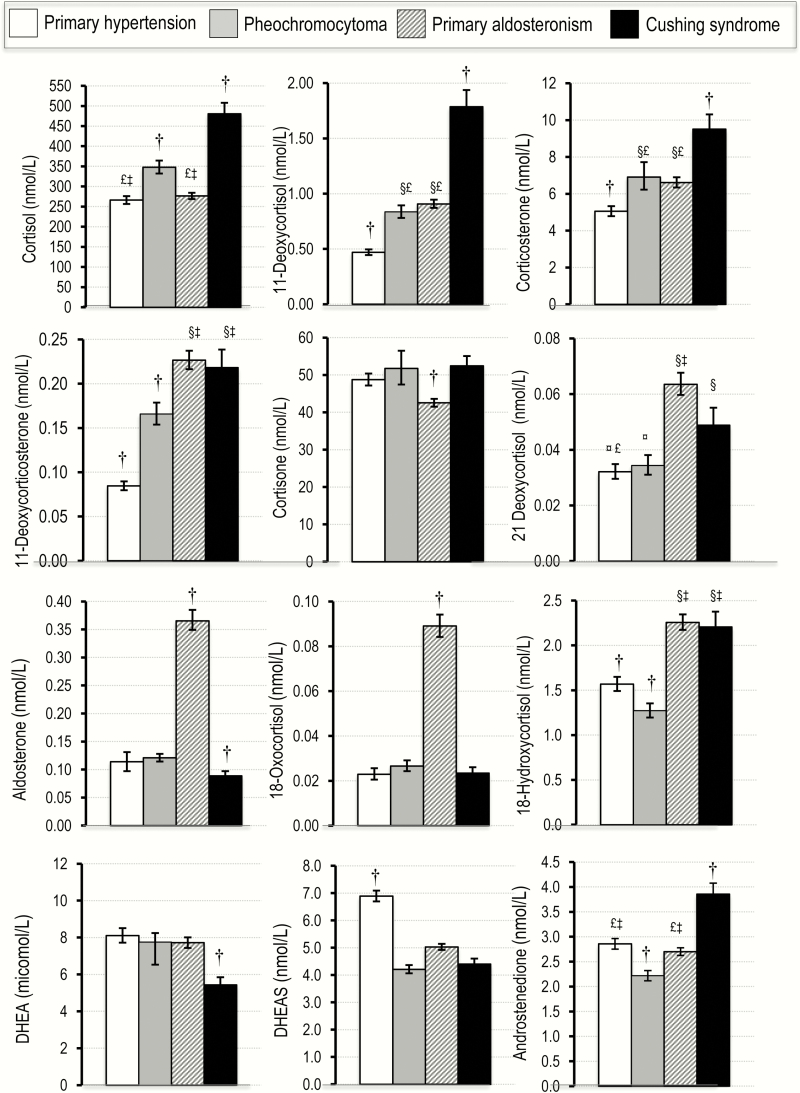
Plasma concentrations for 12 steroids of the 15 steroid panel in patients with pheochromocytoma compared to patients with primary hypertension and patients with Cushing syndrome and primary aldosteronism. Values of steroids are shown as least square means corrected for age and sex and calculated from exponents of logarithmically transformed data (geometric means) with standard errors. ^§^*P *< .05 different from primary hypertension; ^‡^different from pheochromocytoma; ^£^different from Cushing syndrome; ^¤^different from primary aldosteronism; ^†^different from all groups.

With multivariate corrections for age and sex, including generation of least squares means and post hoc analyses using the Tukey HSD test, most of the differences observed for the data in Table 2 were maintained or new differences realized (Fig. 1). In particular, patients with pheochromocytoma showed higher (*P* < .05) plasma concentrations of cortisol, 11-deoxycortisol, corticosterone, and 11-deoxycorticosterone than patients with primary hypertension. In contrast, patients with pheochromocytoma showed lower (*P* < .05) plasma concentrations of androstenedione and DHEAS than patients with primary hypertension.

To put the findings on steroids in PPGL patients in perspective, we also describe patients in whom altered steroid synthesis is the primary culprit for their disorder (ie, patients with hypercortisolism and primary aldosteronism). Patients with Cushing syndrome were distinguished from other groups by elevated (*P* < .05) plasma cortisol, 11-deoxycortisol, and corticosterone, whereas patients with primary aldosteronism were characterized by markedly elevated (*P* < .05) plasma concentrations of aldosterone and 18-oxocortisol compared with all other groups (Fig. 1). Patients with primary aldosteronism and Cushing syndrome both showed similarly higher (*P* < .05) plasma concentrations of 11-deoxycorticosterone and 18-hydroxycortisol than patients with primary hypertension and pheochromocytoma. Patients with pheochromocytoma and primary aldosteronism showed similarly increased (*P* < .05) plasma concentrations of 11-deoxycortisol and corticosterone above concentrations in patients with primary hypertension. However, while cortisol was higher (*P* < .05) in patients with pheochromocytoma than in those with primary aldosteronism, 11-deoxycorticosterone was higher (*P* < .05) in patients with primary aldosteronism than pheochromocytoma. Apart from DHEAS, which showed higher concentrations in patients with primary hypertension than other groups, there were no other steroids remarkably higher in this group than others.

**Figure 2.**
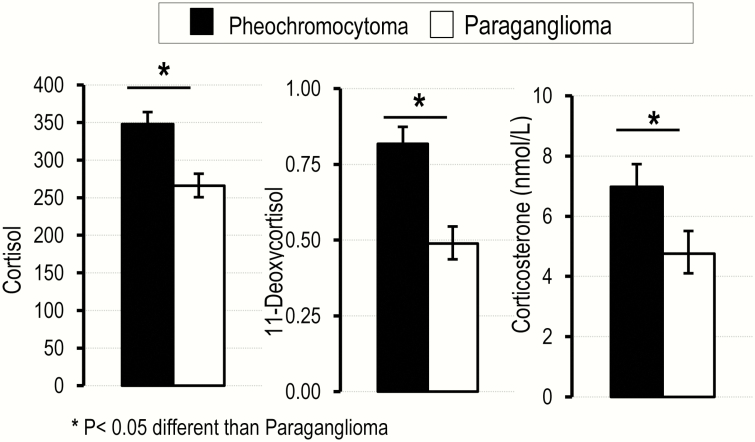
Plasma concentrations for 3 glucocorticoids in patients with pheochromocytoma compared with those with paraganglioma. Values are shown as least square means corrected for age and sex and calculated from exponents of logarithmically transformed data (geometric means) with standard errors. *P < .05 different from paraganglioma.

Similar to the differences with patients with primary hypertension, patients with pheochromocytoma showed higher (*P* < .05) plasma concentrations of cortisol, 11-deoxycortisol, and corticosterone than patients with paraganglioma (Fig. 2). There were no other significant differences in measured plasma steroids between these 2 groups of patients (data not shown).

### Pre- and postoperative differences in steroids

**Figure 3.**
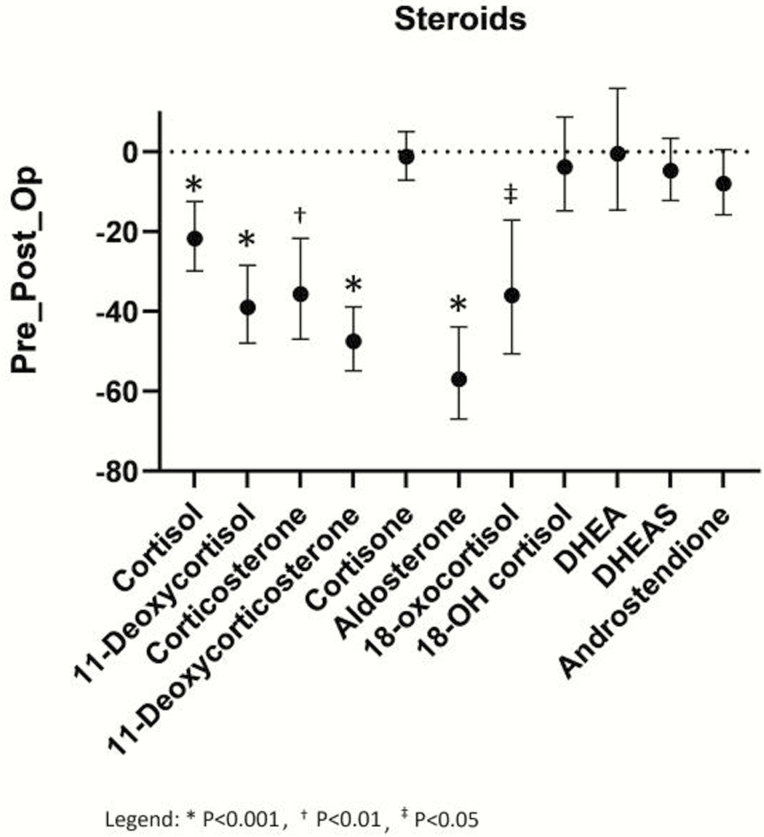
Pre- to postoperative differences in plasma concentrations of 11 steroids after adrenalectomy in patients with pheochromocytoma. Differences in the steroids of interest are shown as geometric means of percentage (%) changes with 95% confidence intervals (CI). Means and confidence intervals were calculated after logarithmic transformation of fold changes and the results retransformed (antilog) as percentages. *P< .001; ^†^*P* < .01; ^‡^*P* < .05.

Following adrenalectomy, patients with pheochromocytoma presented with decreases in several steroids (Fig. 3): cortisol, 11-deoxycortisol, 11-deoxycorticosterone, aldosterone, 18-oxocortisol (*P* < .0001), and corticosterone (*P* = .0002). In contrast, DHEA, androstendione, DHEAS, and 18-hydroxycortisol were not decreased. Pre- to postsurgical changes in steroids were present only in patients with pheochromocytoma. Patients operated for paraganglioma (12 patients) showed no postoperative decreases in steroids, except cortisone (*P* = .0186) (results not shown).

### Indices of catecholamine excess in patients with pheochromocytoma and paraganglioma

Urinary outputs of norepinephrine were not significantly different between patients with paragangliomas and pheochromocytoma (Table 3). In contrast (as further explained in (21)), urinary outputs of normetanephrine, metanephrine, and epinephrine, and plasma concentrations of normetanephrine were lower in patients with paraganglioma than pheochromocytoma.

**Table 3.** Twenty-four hour urinary outputs of catecholamines and metanephrines and plasma concentrations of metanephrines in patients with pheochromocytoma compared to paraganglioma

	Pheochromocytoma	Paraganglioma	
	n = 183	n = 35	*P* value
Urinary NE (nmol/day)	472 (231-1255)^*b*^	388 (175-977)^*a*^	NS
Urinary E (nmol/day)	98 (21-394)	18 (9-28)	<.001
Urinary NMN (nmol/day)	1332 (523-3387)^*b*^	538 (225-1543)^*a*^	.0119
Urinary MN (nmol/day)	534 (94-1761)	86 (51-104)	<.0001
Plasma NMN (nmol/L)	4.26 (1.55-10.7)	2.27 (0.88-6.18)	.0278
Plasma MN (nmol/L)	0.92 (0.17-2.96)	0.17 (0.13-0.22)	<0.0001

Abbreviations: NE, norepinephrine; E, epinephrine; NMN, normetanephrine; MN, metanephrine; NS, not significant. Results are shown as medians and interquartile ranges (25th and 75th); the significance for differences were assessed using Wilcoxon sum ranked-test and are shown as *P* values.

^*a*^N = 29, ^*b*^N = 144.

### Relationships of plasma steroids with indices of catecholamine excess

Among patients with pheochromocytoma, plasma concentrations of 11-deoxycorticosterone and corticosterone were positively (*P* < .02) related with urinary norepinephrine, the summed total of urinary catecholamines (ie, norepinephrine and epinephrine) as well as plasma and urinary free normetanephrine and summed total metanephrines (ie, normetanephrine and metanephrine) in both plasma and urine (Table 4). Weaker relationships were observed for cortisol and 11-deoxycortisol; the former correlated positively (*P* < .05) with the summed total of plasma and urinary metanephrines as well as urinary catecholamines. In contrast, 11-deoxycortisol was only positively (*P* < .01) related to urinary normetanephrine and summed totals of urinary metanephrines. There were no relationships between metanephrine and epinephrine with any of the steroids. Furthermore, for patients with paragangliomas there were no significant relationships of any steroid with any of the various measures of catecholamine excess (results not shown).

**Table 4.** Relationships of plasma steroid concentrations with plasma and urinary metanephrines and urinary catecholamines in patients with pheochromocytoma

	cortisol		11-deoxycortisol		11-deoxycorticosterone		corticosterone	
	r_s_	*P*	r_s_	*P*	r_s_	*P*	r_s_	*P*
**Plasma metanephrines**								
NMN	0.1326	.0744	0.0730	.3271	0.2171	.0032	0.1830	.0134
NMN+MN	0.1874	.0113	0.0869	.2436	0.1916	.0096	0.2170	.0033
**Urinary free metanephrines**								
NMN	0.1487	.0754	0.2166	.0091	0.3472	<.0001	0.2869	.0005
NMN+MN	0.1907	.0216	0.2253	.0064	0.3273	<.0001	0.3160	.0001
**Urinary catecholamines**								
NE	0.1592	.0575	0.1187	.1579	0.2840	.0006	0.2089	.0123
NE+E	0.2101	.0118	0.1371	.1026	0.2589	.0018	0.2426	.0035

Relationships are shown for NMN and NE as well as the sums of both NMN and MN or NE and E according to Spearman’s rank correlation coefficient (r_s_) and significance (*P*).

Abbreviations: NMN, normetanephrine; MN, metanephrine; NE, norepinephrine; E, epinephrine.

## Discussion

This study presents novel data establishing increased circulating glucocorticoids in patients with pheochromocytoma but not paraganglioma. Among the steroids that were increased in patients with pheochromocytoma, 11-deoxycortisol, 11-deoxycorticosterone, and corticosterone showed larger relative increases than cortisol; this pattern has also been observed in clinical and subclinical Cushing syndrome where the same steroids provide larger diagnostic signals than cortisol (20, 22). Moreover, positive relationships were also observed between several glucocorticoids and plasma and urinary markers of catecholamine excess, thus supporting in a clinical setting the concept advanced from preclinical studies by Ehrhart-Bornstein and Bornstein of bidirectional relationships between catecholaminergic and steroidal systems (11, 23, 24).

Although isolated cases of pheochromocytoma in association with Cushing Syndrome have been described, these have involved patients with ectopic ACTH-secreting pheochromocytoma (25-27). These patients presented with both clinical signs of Cushing syndrome and biochemically proven hypercortisolism. Cases of subclinical Cushing in patients with pheochromocytoma have also been noted (28-30). This could be of importance, especially pre- and postoperatively, as severe hypoglycemia has been described postoperatively in 1 of those cases (30). Although hypoglycemia is a frequent postoperative complication in patients with pheochromocytoma, commonly thought to reflect the abrupt postresection fall in circulating catecholamines (31), it is possible that postoperative changes in glucocorticoids could also be a complicating factor. In this context, it was important to investigate whether plasma glucocorticoid concentrations decrease after successful surgery. As we have shown, there was a significant decrease in glucocorticoids and mineralocorticoids after adrenalectomy. Of course, this could also reflect reduced adrenal cortical reserve. On the other hand, there were no postoperative decreases in plasma concentrations of adrenal androgens, suggesting that there was at least postoperative compensation of the zona reticularis in the remaining adrenal.

The findings that only patients with pheochromocytoma and not paraganglioma showed increased circulating concentrations of glucocorticoids and that positive relationships of glucocorticoids to indices of catecholamine excess were only observed for patients with pheochromocytoma suggests that it is the locally produced and not the circulating catecholamines that are responsible for the effect. Since metanephrines are produced by metabolism of catecholamines within tumor cells, the more consistent and stronger positive relationships of metanephrines than catecholamines with circulating glucocorticoids also supports a more likely impact of locally derived rather than circulating catecholamines as the driver responsible for the increases in circulating glucocorticoids. Nevertheless, this cannot be firmly established by the present study. It remains possible that there is some influence of circulating catecholamines that might increase adrenal steroidogenesis in a nonparacrine fashion. Alternatively, the positive relationships between indices of catecholamine excess and increased plasma concentrations of steroids might be indirectly related by way of other mechanisms.

We cannot discriminate between a direct effect of catecholamines on steroid synthesis and an indirect effect mediated through adrenocorticotrophin (ACTH) since we have no data about the latter. Nevertheless, since sustained suppression of ACTH results in reductions of circulating DHEAS (32) the low plasma concentrations of DHEAS in patients with pheochromocytoma appear to be inconsistent with an influence in patients with pheochromocytoma mediated by ACTH. Apart from low ACTH and DHEAS in patients with adrenal Cushing syndrome, other forms of glucocorticoid excess such as subclinical Cushing and primary bilateral macronodular hyperplasia are also associated with reduced plasma concentrations of DHEAS (20, 33-35). Thus, since DHEAS is responsive to ACTH, the lowered plasma concentrations of DHEAS might reflect lower plasma concentrations of ACTH, which may result from feedback inhibition of steroids on the hypothalamic–pituitary–adrenal axis. Although some animal studies have suggested that catecholamines can directly stimulate ACTH secretion (36), this has not been confirmed in humans (37), and there is thus no evidence to implicate circulating catecholamines in the regulation of ACTH secretion.

As with any endogenous compound measured in plasma, circulating concentrations of steroids reflect both their entry into the circulatory compartment and their clearance from that compartment. Thus, it is conceivable that the elevated plasma concentrations of glucocorticoids in patients with pheochromocytoma, rather than reflecting increases in their production might reflect decreased circulatory clearance. Cortisol has a particularly slow plasma clearance, in part due to the high proportion of the steroid that is bound to transcortin; thus, it is possible that differences in binding to transcortin could also decrease the clearance of glucocorticoids and through this action increase plasma concentrations. Nevertheless (as further detailed in (21)) it seems unlikely that the higher plasma concentrations of glucocorticoids, lower concentrations of DHEAS and unchanged concentrations of most other steroids in patients with pheochromocytoma could reflect divergent effects on clearance or protein binding rather than an effect on production.

Although elevated plasma concentrations of the 3 glucocorticoids—cortisol, 11-deoxycortisol, and corticosterone—in patients with pheochromocytoma were nowhere close to the higher concentrations in patients with Cushing syndrome, they were similar, or even higher in the case of cortisol, to concentrations in primary aldosteronism. Of relevance to these findings are those of Arlt et al. (38), who showed using mass spectrometry–based urinary steroid profiling that patients with primary aldosteronism were characterized by increased urinary outputs of not only free cortisol, but also tetrahydro-11-deoxycorticosterone and tetrahydrocortisone. The last 2 steroid metabolites are respectively produced from 11-deoxycorticosterone and corticosterone. Thus, the present findings in plasma support the earlier findings in urine. Lack of increase in plasma cortisol in the present study (as further detailed in (21)), but increased urinary free cortisol in the earlier study of Arlt et al. (38) is easily explained by the inferiority of morning plasma cortisol compared with the other 3 plasma corticosteroids and urinary free cortisol for assessing hypercortisolism. Since production of excess cortisol in primary aldosteronism is now recognized as a potential contributing factor to the excess morbidity of primary aldosteronism (39, 40), it seems possible that the same may apply to patients with pheochromocytoma.

The strengths of this study include the multicenter inclusion of a large number of patients with different forms of adrenal hypertension as well as large numbers of patients with primary hypertension. One limitation, in addition to the aforementioned lack of data on ACTH, relates to lack of measurements of 24 hour urinary free cortisol. Also, we had a high proportion of females in the group with Cushing syndrome, as is expected in such patients (41). Although we used multivariate analyses to correct for such influences, it remains possible that this may have been insufficient for some comparisons. Nevertheless, such differences along with the sex imbalance in the patients with Cushing syndrome are not relevant to the higher plasma concentrations of glucocorticoids in patients with PPGLs compared with hypertensive groups.

Pheochromocytomas are among the most life threatening of all endocrine diseases, with elevated morbidity and mortality, especially if undiagnosed. The diagnosis represents a challenge, due partly to lack of specificity of most clinical signs and symptoms. The cardiovascular complications are mainly secondary to excessive secretion of catecholamines from tumors. However, the present data raise the possibility that glucocorticoids might also contribute to the cardiovascular and metabolic complications caused by pheochromocytomas. This supports the concept that altered steroid concentrations in patients with pheochromocytoma may have clinical consequences.

## Abbreviations

ACTH: adrenocorticotrophin
CI: confidence interval
DHEA: dehydroepiandrosterone
DHEAS: dehydroepiandrosterone sulfate
LC-MS/MS: liquid chromatography tandem mass spectrometry
PNMT: phenylethanolamine-N-methyltransferase
PPGL: pheochromocytoma and paraganglioma
BP: blood pressure
PHT: primary hypertensives

## Additional Information

***Disclosure Summary:*** The authors have nothing to disclose. The authors have no conflicts of interest.

***Data Availability:*** The datasets generated during and/or analyzed during the current study are not publicly available but are available from the corresponding author on reasonable request.

## References

[1] Lenders JWM, Eisenhofer G Update on modern management of pheochromocytoma and paraganglioma. Endocrinol Metab (Seoul). 2017;32(2):152-161. 28685506 10.3803/EnM.2017.32.2.152 PMC5503859

[2] Isidori AM, Graziadio C, Paragliola RM, et al.; ABC Study Group The hypertension of Cushing’s syndrome: controversies in the pathophysiology and focus on cardiovascular complications. J Hypertens. 2015;33(1):44-60. 25415766 10.1097/HJH.0000000000000415 PMC4342316

[3] Wurtman RJ, Axelrod J Adrenaline synthesis: control by the pituitary gland and adrenal glucocorticoids. Science. 1965;150(3702):1464-1465. 4285299 10.1126/science.150.3702.1464

[4] Qin N, de Cubas AA, Garcia-Martin R, et al. Opposing effects of HIF1α and HIF2α on chromaffin cell phenotypic features and tumor cell proliferation: Insights from MYC-associated factor X. Int J Cancer. 2014;135(9):2054-2064. 24676840 10.1002/ijc.28868

[5] Ehrhart-Bornstein M, Bornstein SR, Güse-Behling H, et al. Sympathoadrenal regulation of adrenal androstenedione release. Neuroendocrinology. 1994;59(4):406-412. 8202222 10.1159/000126685

[6] Ehrhart-Bornstein M, Bornstein SR, González-Hernández J, Holst JJ, Waterman MR, Scherbaum WA Sympathoadrenal regulation of adrenocortical steroidogenesis. Endocr Res. 1995;21(1-2):13-24. 7588375 10.3109/07435809509030417

[7] Ehrhart-Bornstein M, Hilbers U Neuroendocrine properties of adrenocortical cells. Horm Metab Res. 1998;30(6-7):436-439. 9694576 10.1055/s-2007-978911

[8] Lefebvre H, Prévost G, Louiset E Autocrine/paracrine regulatory mechanisms in adrenocortical neoplasms responsible for primary adrenal hypercorticism. Eur J Endocrinol. 2013;169(5):R115-R138. 23956298 10.1530/EJE-13-0308

[9] Güse-Behling H, Ehrhart-Bornstein M, Bornstein SR, Waterman MR, Scherbaum WA, Adler G Regulation of adrenal steroidogenesis by adrenaline: expression of cytochrome P450 genes. J Endocrinol. 1992;135(2):229-237. 1335470 10.1677/joe.0.1350229

[10] Ehrhart-Bornstein M, Bornstein SR, Scherbaum WA Sympathoadrenal system and immune system in the regulation of adrenocortical function. Eur J Endocrinol. 1996;135(1):19-26. 8765968 10.1530/eje.0.1350019

[11] Ehrhart-Bornstein M, Bornstein SR Cross-talk between adrenal medulla and adrenal cortex in stress. Ann N Y Acad Sci. 2008;1148(1):112-117. 19120098 10.1196/annals.1410.053

[12] Lenders JW, Duh QY, Eisenhofer G, et al.; Endocrine Society Pheochromocytoma and paraganglioma: an endocrine society clinical practice guideline. J Clin Endocrinol Metab. 2014;99(6):1915-1942. 24893135 10.1210/jc.2014-1498

[13] Funder JW, Carey RM, Mantero F, et al. The management of primary aldosteronism: case detection, diagnosis, and treatment: an endocrine society clinical practice guideline. J Clin Endocrinol Metab. 2016;101(5):1889-1916. 26934393 10.1210/jc.2015-4061

[14] Nieman LK, Biller BM, Findling JW, et al. The diagnosis of Cushing’s syndrome: an endocrine society clinical practice guideline. J Clin Endocrinol Metab. 2008;93(5):1526-1540. 18334580 10.1210/jc.2008-0125 PMC2386281

[15] Peitzsch M, Dekkers T, Haase M, et al. An LC-MS/MS method for steroid profiling during adrenal venous sampling for investigation of primary aldosteronism. J Steroid Biochem Mol Biol. 2015;145(1):75-84. 25312486 10.1016/j.jsbmb.2014.10.006

[16] Peitzsch M, Prejbisz A, Kroiß M, et al. Analysis of plasma 3-methoxytyramine, normetanephrine and metanephrine by ultraperformance liquid chromatography-tandem mass spectrometry: utility for diagnosis of dopamine-producing metastatic phaeochromocytoma. Ann Clin Biochem. 2013;50(Pt 2):147-155. 23512172 10.1258/acb.2012.012112

[17] Peitzsch M, Pelzel D, Glöckner S, et al. Simultaneous liquid chromatography tandem mass spectrometric determination of urinary free metanephrines and catecholamines, with comparisons of free and deconjugated metabolites. Clin Chim Acta. 2013;418(4):50-58. 23313054 10.1016/j.cca.2012.12.031

[18] Eisenhofer G, Peitzsch M, Kaden D, et al. Reference intervals for LC-MS/MS measurements of plasma free, urinary free and urinary acid-hydrolyzed deconjugated normetanephrine, metanephrine and methoxytyramine. Clin Chim Acta. 2019;490(3):46-54. 30571948 10.1016/j.cca.2018.12.019

[19] Eisenhofer G, Peitzsch M, Kaden D, et al. Reference intervals for plasma concentrations of adrenal steroids measured by LC-MS/MS: impact of gender, age, oral contraceptives, body mass index and blood pressure status. Clin Chim Acta. 2017;470(7):115-124. 28479316 10.1016/j.cca.2017.05.002 PMC5504266

[20] Masjkur J, Gruber M, Peitzsch M, et al. Plasma steroid profiles in subclinical compared with overt adrenal Cushing syndrome. J Clin Endocrinol Metab. 2019;104(10):4331-4340. 30977834 10.1210/jc.2018-02349

[21] Constantinescu G, Langton K, Conrad C Glucocorticoid excess in patients with pheochromocytoma compared with paraganglioma and other forms of hypertension. Opara Repository. Deposited June 15, 2020 10.25532/OPARA-85 PMC7413598 32609829

[22] Eisenhofer G, Masjkur J, Peitzsch M, et al. Plasma steroid metabolome profiling for diagnosis and subtyping patients with Cushing syndrome. Clin Chem. 2018;64(3):586-596. 29208661 10.1373/clinchem.2017.282582

[23] Ehrhart-Bornstein M, Hinson JP, Bornstein SR, Scherbaum WA, Vinson GP Intraadrenal interactions in the regulation of adrenocortical steroidogenesis. Endocr Rev. 1998;19(2):101-143. 9570034 10.1210/edrv.19.2.0326

[24] Schinner S, Bornstein SR Cortical-chromaffin cell interactions in the adrenal gland. Endocr Pathol. 2005;16(2):91-98. 16199893 10.1385/ep:16:2:091

[25] Oh HC, Koh JM, Kim MS, et al. A case of ACTH-producing pheochromocytoma associated with pregnancy. Endocr J. 2003;50(6):739-744. 14709846 10.1507/endocrj.50.739

[26] Nijhoff MF, Dekkers OM, Vleming LJ, Smit JW, Romijn JA, Pereira AM ACTH-producing pheochromocytoma: clinical considerations and concise review of the literature. Eur J Intern Med. 2009;20(7):682-685. 19818286 10.1016/j.ejim.2009.08.002

[27] Langton K, Gruber M, Masjkur J, et al. Hypertensive crisis in pregnancy due to a metamorphosing pheochromocytoma with postdelivery Cushing’s syndrome. Gynecol Endocrinol. 2018;34(1):20-24. 28937294 10.1080/09513590.2017.1379497

[28] Finkenstedt G, Gasser RW, Höfle G, et al. Pheochromocytoma and sub-clinical Cushing’s syndrome during pregnancy: diagnosis, medical pre-treatment and cure by laparoscopic unilateral adrenalectomy. J Endocrinol Invest. 1999;22(7):551-557. 10475154 10.1007/BF03343608

[29] Takizawa N, Muguruma K, Sasano H Pheochromocytoma and subclinical Cushing’s syndrome with focal adrenocortical hyperplasia. Int J Urol. 2011;18(7):548-549. 21481011 10.1111/j.1442-2042.2011.02759.x

[30] Kastelan D, Ravic KG, Cacic M, et al. Severe postoperative hypoglycemia in a patient with pheochromocytoma and preclinical Cushing’s syndrome. Med Sci Monit. 2007;13(3):CS34-CS37. 17325639

[31] Araki S, Kijima T, Waseda Y, et al. Incidence and predictive factors of hypoglycemia after pheochromocytoma resection. Int J Urol. 2019;26(2):273-277. 30467902 10.1111/iju.13864

[32] McKenna TJ, Fearon U, Clarke D, Cunningham SK A critical review of the origin and control of adrenal androgens. Baillieres Clin Obstet Gynaecol. 1997;11(2):229-248. 9536209 10.1016/s0950-3552(97)80035-1

[33] Yener S, Yilmaz H, Demir T, Secil M, Comlekci A DHEAS for the prediction of subclinical Cushing’s syndrome: perplexing or advantageous? Endocrine. 2015;48(2):669-676. 25146553 10.1007/s12020-014-0387-7

[34] Hannah-Shmouni F, Berthon A, Faucz FR, et al. Mass spectrometry-based steroid profiling in primary bilateral macronodular adrenocortical hyperplasia. Endocr Relat Cancer. 2020;27(7):403-413. 32348959 10.1530/ERC-20-0102 PMC7354003

[35] Dennedy MC, Annamalai AK, Prankerd-Smith O, et al. Low DHEAS: a sensitive and specific test for the detection of subclinical hypercortisolism in adrenal incidentalomas. J Clin Endocrinol Metab. 2017;102(3):786-792. 27797672 10.1210/jc.2016-2718

[36] Mezey E, Reisine TD, Palkovits M, Brownstein MJ, Axelrod J Direct stimulation of beta 2-adrenergic receptors in rat anterior pituitary induces the release of adrenocorticotropin in vivo. Proc Natl Acad Sci U S A. 1983;80(21):6728-6731. 6314339 10.1073/pnas.80.21.6728 PMC391244

[37] Eisenhofer G, Goldstein DS, Stull RW, Gold PW, Keiser HR, Kopin IJ Dissociation between corticotrophin and catecholamine responses to isoprenaline in humans. Clin Exp Pharmacol Physiol. 1987;14(4):337-341. 2822312 10.1111/j.1440-1681.1987.tb00980.x

[38] Arlt W, Lang K, Sitch AJ, et al. Steroid metabolome analysis reveals prevalent glucocorticoid excess in primary aldosteronism. JCI Insight. 2017;2(8):e93136. 10.1172/jci.insight.93136 PMC5396526 28422753

[39] Adolf C, Köhler A, Franke A, et al. Cortisol excess in patients with primary aldosteronism impacts left ventricular hypertrophy. J Clin Endocrinol Metab. 2018;103(12):4543-4552. 30113683 10.1210/jc.2018-00617

[40] Akehi Y, Yanase T, Motonaga R, et al.; Japan Primary Aldosteronism Study Group High prevalence of diabetes in patients with primary aldosteronism (PA) associated with subclinical hypercortisolism and prediabetes more prevalent in bilateral than unilateral PA: a large, multicenter cohort study in Japan. Diabetes Care. 2019;42(5):938-945. 31010944 10.2337/dc18-1293

[41] Lindholm J, Juul S, Jørgensen JO, et al. Incidence and late prognosis of Cushing’s syndrome: a population-based study. J Clin Endocrinol Metab. 2001;86(1):117-123. 11231987 10.1210/jcem.86.1.7093

